# Recurrent hypoglycaemia promotes cardiomyopathy and cardiac vulnerability in a rodent model of type 1 diabetes

**DOI:** 10.1007/s00125-025-06574-5

**Published:** 2025-11-10

**Authors:** Calum Forteath, Heather J. Merchant, Cyril Kocherry, Colin E. Murdoch, Jennifer Kerr, Jennifer R. Gallagher, Mark L. Evans, Bernard Thorens, Ulrik Pedersen-Bjergaard, Bastiaan E. de Galan, Rory J. McCrimmon, Alison D. McNeilly

**Affiliations:** 1 Diabetes, Endocrinology and Reproductive Biology, School of Medicine, University of Dundee, Dundee, UK; 2 Cardiovascular Research, School of Medicine, University of Dundee, Dundee, UK; 3 Institute of Metabolic Science Metabolic Research Laboratories, University of Cambridge, Cambridge, UK; 4 Centre for Integrative Geonomics, University of Lausanne, Lausanne, Switzerland; 5 Department of Clinical Medicine, Faculty of Health and Medical Sciences, University of Copenhagen, Copenhagen, Denmark; 6 Department of Endocrinology and Nephrology, Nordsjællands University Hospital Hillerød, Hillerød, Denmark; 7 Department of Internal Medicine, Radboud University Medical Centre, Nijmegen, the Netherlands; 8 Department of Internal Medicine, Maastricht University Medical Centre, Maastricht, the Netherlands; 9 Cardiovascular Research Institute Maastricht (CARIM), School for Cardiovascular Diseases, Maastricht University, Maastricht, the Netherlands

**Keywords:** Cardiovascular disease, Dilated cardiomyopathy, Hypoglycaemia, Left ventricle, Microvascular dysfunction, Type 1 diabetes

## Abstract

**Aims/hypothesis:**

CVD remains the leading cause of mortality in individuals with type 1 diabetes over the age of 40 years. Although intensive insulin therapy lowers chronic hyperglycaemia and improves cardiovascular outcomes, it also increases the frequency of hypoglycaemic episodes, an emerging but poorly understood contributor to CVD risk. The mechanisms by which recurrent hypoglycaemia exacerbates cardiovascular pathology in type 1 diabetes are unknown.

**Methods:**

Using a C57BL/J streptozocin-induced male mouse model of type 1 diabetes, combined with detailed physiological and molecular assessments, we investigated the impact of recurrent hypoglycaemia (<3.0 mmol/l) on cardiovascular structure and function using laser Doppler imaging with iontophoresis and ultrasound imaging.

**Results:**

Type 1 diabetes induces significant microvascular endothelial dysfunction, which is worsened by recurrent hypoglycaemia. Chronic exposure to hypoglycaemia (60 episodes over 20 weeks) resulted in compensatory shifts in cardiac haemodynamics, which in type 1 diabetic mice but not non-diabetic mice resulted in early dilated cardiomyopathy. In both type 1 diabetic and non-diabetic mice, recurrent hypoglycaemia resulted in impaired systolic function during a subsequent hypoglycaemic challenge, indicating increased cardiac vulnerability despite any compensatory changes. Transcriptomic profiling of left ventricular tissue revealed that recurrent hypoglycaemia induces distinct gene expression changes involving ion homeostasis, repolarisation dynamics and microvascular signalling—molecular alterations characteristic of early diabetic cardiomyopathy.

**Conclusions/interpretation:**

These findings provide the first in vivo evidence that recurrent hypoglycaemia synergises with hyperglycaemia to accelerate microvascular dysfunction and adverse cardiac remodelling in type 1 diabetes. This work identifies a novel mechanistic link between hypoglycaemia and diabetic heart disease, underscoring the need for therapeutic strategies that mitigate glycaemic variability without increasing the hypoglycaemic burden.

**Data availability:**

Transcriptomic data are available as supplementary data.

**Graphical Abstract:**

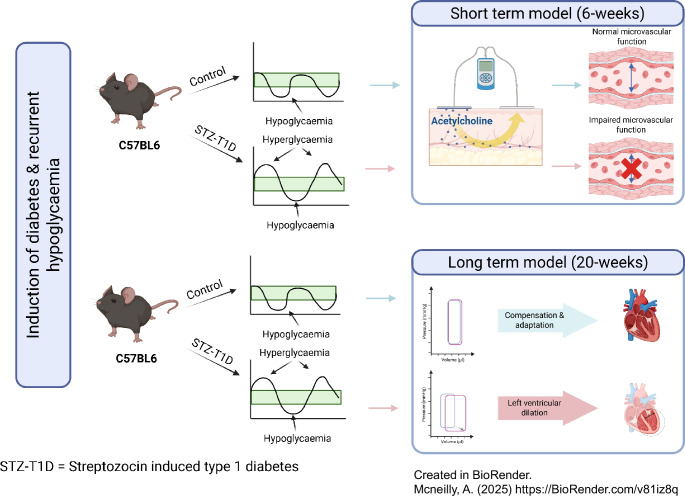

**Supplementary Information:**

The online version contains peer-reviewed but unedited supplementary material available at 10.1007/s00125-025-06574-5.


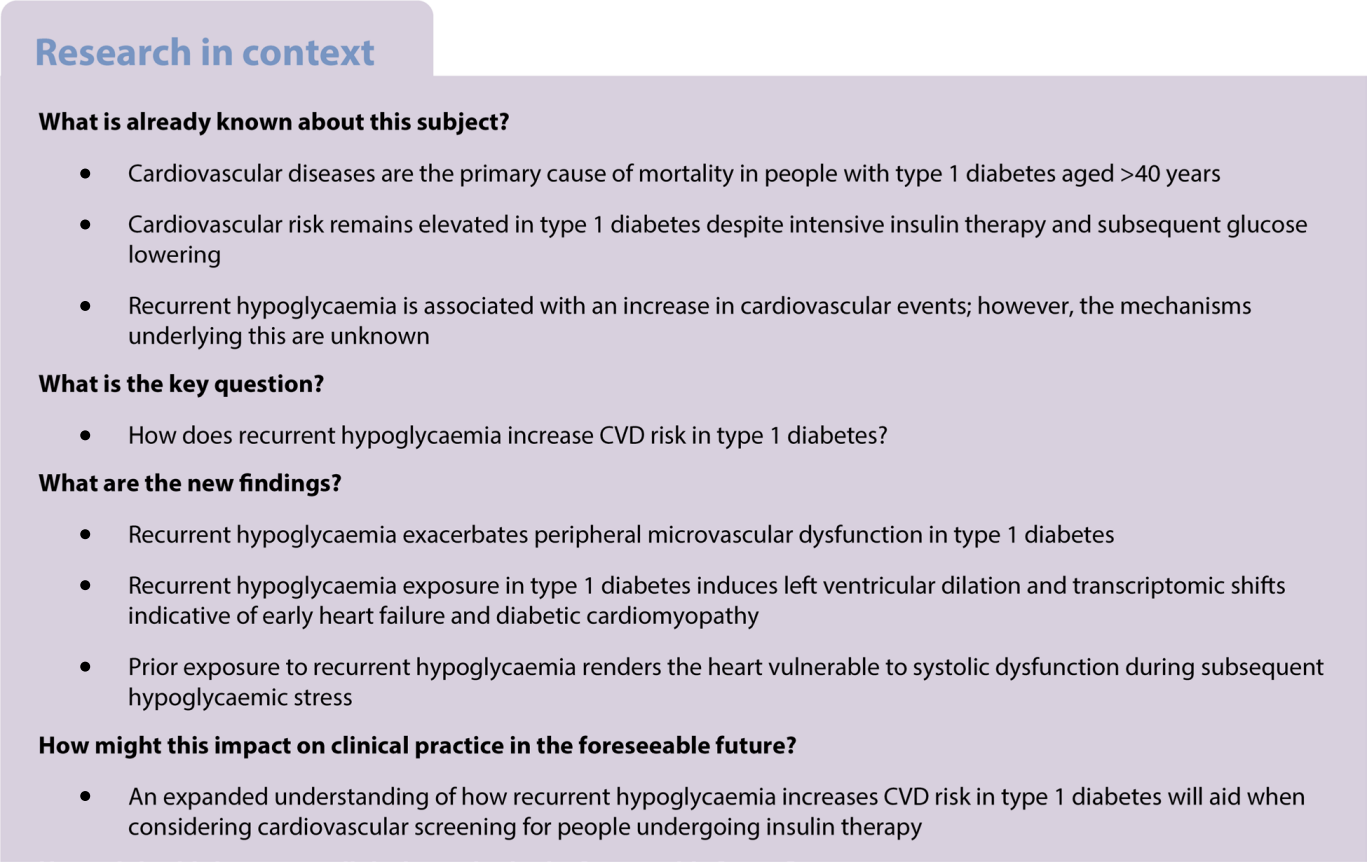


## Introduction

Diabetes is a global health emergency, with recent estimates based on current trends suggesting that global prevalence will reach 1.3 billion worldwide by 2050 [1]. It is estimated that the management of diabetes and its complications will cost at least US$2.1 trillion annually by the year 2030 and contribute to the deaths of 1.59 million people in 2025 [2, 3]. The two major subtypes of diabetes are type 1 and type 2 diabetes, with type 1 diabetes accounting for up to 10% of all cases, depending on ethnicity, and resulting from the autoimmune destruction of pancreatic beta cells. Both conditions, inadequately treated, lead to chronic hyperglycaemia, which in turn increases the risk of micro- and macrovascular disease. In people with type 2 diabetes and those with type 1 diabetes aged >40 years, CVD is the greatest cause of morbidity and mortality [4]. Chronic hyperglycaemia is a well-established, independent risk factor for cardiovascular complications in both type 1 and type 2 diabetes. Hyperglycaemia is associated with increased mitochondrial dysfunction, elevated production of reactive oxygen species (ROS) and inflammation [5, 6], which contribute to tissue damage [7]. Chronic hyperglycaemia may also lead to cardiomyopathy and cardiac autonomic neuropathy (CAN), directly increasing the risk of cardiac-related death [8].

Lowering median blood glucose levels in people with type 1 diabetes has been shown to improve, but not reverse, micro- and macrovascular complications, including CVD [9]. One potential explanation for this is the difficulty in achieving recommended glucose targets without experiencing hypoglycaemia [10]. Hypoglycaemia is common in type 1 diabetes, with most population studies suggesting a mean incidence of two episodes per week [11]. A recent analysis of pooled data from 84 clinical trials with 39,373 participants found evidence of an association between hypoglycaemic episodes of any kind in the previous 10 days and death, acute CVD and retinal disorders in both type 1 and type 2 diabetes, with rate ratios ranging from 1.32 (*p*=0.017) to 2.68 (*p*<0.0001) [12]. In human studies, experimental hypoglycaemia has been shown to induce an acute inflammatory response [13, 14] associated with increased oxidative stress [15] and impaired nitric oxide (NO)-dependent vasodilation [16]. Acute hypoglycaemia in humans also results in monocyte mobilisation, increased platelet reactivity, promotion of the interaction between platelets and proinflammatory monocytes, and potentiation of the subsequent immune response to endotoxin [17]. The proinflammatory response following hypoglycaemia is sustained for at least 1 week in people with type 1 diabetes [14]. Interestingly, in rodents, chronic hyperglycaemia and post-hypoglycaemic hyperglycaemia act synergistically to induce oxidative stress and cellular damage [18]. This led us to hypothesise that recurrent hypoglycaemia in type 1 diabetes exacerbates the effect of chronic exposure to high glucose on microvascular and cardiac function. The series of studies presented here tests this hypothesis in a mouse model of type 1 diabetes.

## Methods

### Experimental animals

Male C57BL6 mice (20–25 g, aged 10 weeks,* n*=110 total; Charles River Laboratories, UK) were acclimatised for 2 weeks prior to induction of experimental diabetes via streptozocin (STZ) injection. Animals were housed, typically four animals per cage with additional bedding material for enrichment, in a temperature-controlled (19–23°C) and humidity-controlled (45–60%) environment with a 12 h light/dark cycle at the University of Dundee. Food and water were provided ad libitum unless otherwise stated (see ‘induction of hypoglycaemia’). Experiments were performed in line with UK Home Office guidance on the operation of the Animals (Scientific Procedures) Act 1986 under the auspices of project licence PP2258914. Study guidelines were approved by the University of Dundee Ethics Committee and the named veterinary surgeon.

### Mouse model of type 1 diabetes

Mice were randomly assigned to receive either a single dose of STZ (Sigma Aldrich; 150 mg/kg, i.p.) in HBSS (Gibco UK) to induce chemical pancreatic beta cell destruction akin to chronic type 1 diabetes (STZ-T1D mice), or an equivalent volume of STZ-free HBSS (i.p.) (control mice) [19]. Confirmation of hyperglycaemia onset was performed by tail vein blood glucose assessment using a CONTOUR 85726688 hand-held glucometer 72 h and 7 days post-injection with STZ. Blood glucose levels ≥16 mmol/l were accepted as indicating insulin-deficient diabetes.

### Insulin replacement

To prevent continuous body weight loss and tissue catabolism and to maintain a moderate degree of hyperglycaemia, type 1 diabetic mice had subcutaneous insulin pellets (LinBit, LinShin, Canada) surgically implanted using half of the recommended dose (~0.05 U/kg per day). Non-hyperglycaemic control animals underwent ‘sham’ surgery. Details of animal weights and glucose profiles following insulin implantation can be found in ESM Table 1.

### Induction of hypoglycaemia

**Fig. 1:**
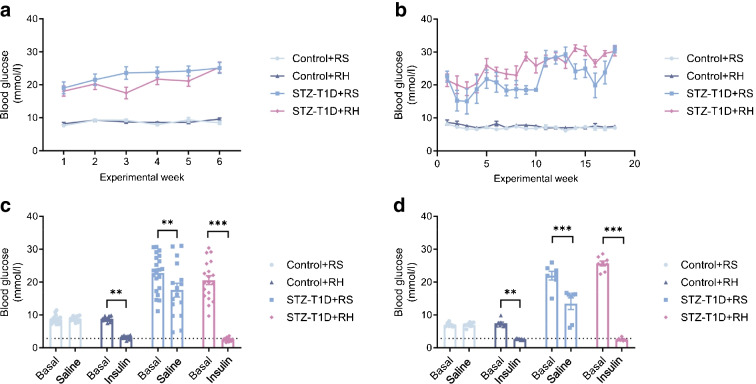
Glucose profiles and moderate (‘level 2’) recurrent hypoglycaemia in 6 week and 20 week experimental mice models. (**a**, **b**) Basal blood glucose over 6 weeks (**a**) and 20 weeks (**b**), showing that STZ administration in mice successfully induced chronic hyperglycaemia that persisted throughout the study periods vs control mice receiving STZ-free buffer (vehicle). (**c**, **d**) Mean lowest blood glucose nadirs following injections of saline (+RS) or insulin (+RH) over 6 weeks (**c**) and 20 weeks (**d**). The dotted line in (**c**) and (**d**) intersects the y-axis at 2.8 mmol/l, indicating that the target range of level 2 hypoglycaemia was reached in both recurrent hypoglycaemia groups. Data are presented as mean ± SEM. Statistical significance determined by two-way ANOVA with Tukey’s post hoc analysis: **p<0.01, ***p<0.001. (**a**, **c**) 6 week cohort, *n*=16–21 mice per group; (**b**, **d**) 20 week cohort, *n*=7–8 mice per group

Mice were subjected to three episodes per week of insulin-induced (Actrapid, Novo Nordisk, s.c.) hypoglycaemia (target glucose 2.0–3.0 mmol/l) for 6 weeks (short-term study) or 20 weeks (long-term study) (electronic supplementary material [ESM] Fig. 1). Food was withdrawn on measurement of resting blood glucose in both insulin groups (STZ-T1D+RH, Control+RH) and saline groups (STZ-T1D+RS, Control+RS) and returned immediately post hypoglycaemia. The insulin dose was based on basal blood glucose measurements and body weight prior to injection [19]. Control mice received volume-matched saline (s.c.; 0.9% wt/vol. NaCl). Blood glucose levels were recorded at 45 and 90 min post injection. Animals whose blood glucose levels dropped to <2.0 mmol/l within 90 min were given access to water-softened chow. Four animals required 50 mg/kg glucose in saline (i.p.) to aid in their recovery (four occasions out of a total of 2826 hypoglycaemic events).

### Microvascular blood flow assessment

Peripheral microvascular function provides a surrogate measure for predicting cardiovascular risk [20]. Using non-invasive imaging under light, inhaled (1.5% isoflurane) anaesthesia, we assessed skin microvascular blood flow by laser Doppler imaging (MOORld12-vr, Moor Instruments) coupled with transdermal application of vasoactive compounds (acetylcholine [Ach] and phenylephrine) via iontophoresis, as described previously [21].

### Pressure–volume loops

Pressure–volume (PV) loops were assessed via direct catheterisation of the left ventricle using a 1.4F Transonics PV catheter [22]. Mice were placed on a heated surgical plate to maintain body temperature at 37°C and ensure a stable heart rate under anaesthesia (2% isoflurane). Initially, the right carotid artery was carefully exposed, and a PV catheter was inserted into the right carotid artery to measure arterial blood pressure. The catheter was then advanced retrogradely via the aortic arch into the left ventricle to assess PV loops, which allow for a full assessment of cardiac haemodynamics throughout the cardiac cycle. Cardiac function was analysed during two key phases—systole (contraction) and diastole (relaxation)—with both pressure and volume being simultaneously recorded in vivo. This process produces a distinct loop that simulates the cardiac cycle and the interplay between ventricular pressure and volume. Cardiac load-independent systolic and diastolic measurements were assessed under occlusion PV loops, achieved by occluding the inferior vena cava and modifying preload conditions on the heart. Cardiac parameters were measured using LabChart Software (ADInstruments, Oxford, UK).

### Echocardiography

Cardiac function was assessed longitudinally at five time points (ESM Fig. 1) using ultrasound echocardiography (Fujifilm VisualSonics VEVOF2, UHF46x Transducer [46–20 MHz] under isoflurane anaesthesia [1.5% in room air, Kent Scientific SomnoSuite Apparatus]). Briefly, mice were anaesthetised and placed in a supine position on a thermoregulatory heating board with paws connected to ECG electrode pads using Transpore surgical tape. A digital thermometer was inserted rectally to provide feedback to the thermoregulatory board for maintaining thermoneutrality at 37°C. Hair was removed from the abdomen using depilatory cream (Veet), and the abdomen was thoroughly washed with clean water to improve image quality. Warmed ultrasound imaging gel was applied to the abdomen and 2D images were acquired using 5 s recordings at the papillary muscle depth within the left ventricular long axis (B-mode) using the ‘Cardiac’ system preset, ensuring thermoneutrality and a physiological heart rate (>400 beats per minute [bpm]) throughout the recordings. Image analysis was performed blinded on three to six cycles using Fujifilm VisualSonics Vevo LAB software version 8 by a trained technician (CK) who was blinded to treatment group, and independently reassessed by CF (20% of randomly assigned image sets).

Cardiac function was also assessed under hypoglycaemic conditions to determine how the heart adapts to acute hypoglycaemia after recurrent exposure to hypoglycaemia. Mice were given insulin (s.c.) and returned to fresh home cages without food until they became hypoglycaemic (blood glucose <3.0 mmol/l, determined by glucometer readings of tail vein blood). Animals were anaesthetised and prepared (as above), and images were collected within 15 min of anaesthetic induction. Blood glucose was monitored throughout the procedure (<5µl tail vein blood) to ensure the animals were hypoglycaemic during data collection. Animals were provided with soft chow and monitored during recovery.

### Tissue collection

Following PV measurements, animals were immediately culled via isoflurane anaesthesia overdose and blood was obtained by cardiac puncture, transferred to an EDTA-coated microtube and stored on ice until centrifugation at 1975 *g* for 30 min at 4°C. Plasma was separated and stored at −80°C until further assessment. Perfusion with 5 mmol/l KCl was performed at a pressure of 100 mmHg by direct infusion into the left ventricle through a needle connected to a KCl reservoir, secured 1 m above the animal, to arrest the heart in diastole. Tissues were carefully isolated, gently rinsed in clean PBS, dabbed dry and weighed using a fine balance. Afterwards, tissue was stored in 4% paraformaldehyde or snap-frozen for storage at −80°C.

### Left ventricle RNA-seq

Left ventricle tissue was dissected from the whole heart and RNA extracted using the TRIzol method (see ESM Methods, ‘Tissue dissection and RNA extraction’ for details). RNA-seq was performed by GENEWIZ, Azenta Life Sciences (see ESM Methods, ‘RNA library preparation and NovaSeq sequencing’ for details). In short, RNA was quantified using a Qubit 4.0 Fluorometer, and libraries were prepared with the NEBNext Ultra II RNA Library Prep Kit and sequenced on an Illumina NovaSeq 6000 instrument. Sequence reads were mapped to the ENSMBL *Mus musculus* reference genome, and the RStudio package DESeq2 was used for gene expression comparisons [23]. The Wald test was used to generate *p* values and log_2_ fold changes. Downstream Gene Ontology (GO) and Kyoto Encyclopedia of Genes and Genomes (KEGG) analysis was performed using RStudio package clusterProfiler [24, 25] and the data visualised using RStudio package ggplot2 (autumn 2023) (see ESM Methods, ‘RNA-seq data analysis and visualisation’ for details).

### Materials

Unless otherwise stated, materials were sourced from Merck-Sigma.

### Statistical analysis

Data were analysed using GraphPad Prism version 10.2.2 and IBM SPSS version 18. All group data are reported as mean ± SEM with group size (*n*) specified for each experiment. Four animals were excluded from analysis due to fitting following insulin injection. The Shapiro–Wilk test was used to determine whether the data were normally distributed (α >0.05). Two-way ANOVA with fixed factors of diabetes status and hypoglycaemia condition was used to determine the main effects of both type 1 diabetes and recurrent hypoglycaemia and any interaction between the two. A mixed-effects ANOVA with fixed factors of diabetic status and acute and recurrent hypoglycaemia was used to determine the effects of acute vs recurrent hypoglycaemia and glycaemic background on echocardiography studies during hypoglycaemia. Post hoc analysis was performed using Tukey’s multiple comparisons test. Statistical significance was set at *p*<0.05.

## Results

### Recurrent hypoglycaemia in mice

STZ administration followed by partial insulin replacement, successfully induced moderate hyperglycaemia in mice (Fig. 1a, b). Insulin-induced hypoglycaemia was achieved in both hyperglycaemic mice (STZ-T1D+RH: 2.51 ± 0.12 mmol/l [6 weeks], 2.60 ± 0.07 mmol/l [20 weeks]) and control mice (Control+RH: 2.94 ± 0.12 mmol/l [6 weeks], 2.50 ± 0.15 mmol/l [20 weeks]) (Fig. 1c, d). A small but statistically significant drop in blood glucose was observed following saline injection in STZ-T1D animals (STZ-T1D+RS blood glucose nadir 17.57 ± 2.08 mmol/l [6 weeks], 13.43 ± 1.80 mmol/l [20 weeks]).

### Recurrent hypoglycaemia worsens microvascular endothelial dysfunction in diabetes

**Fig. 2:**
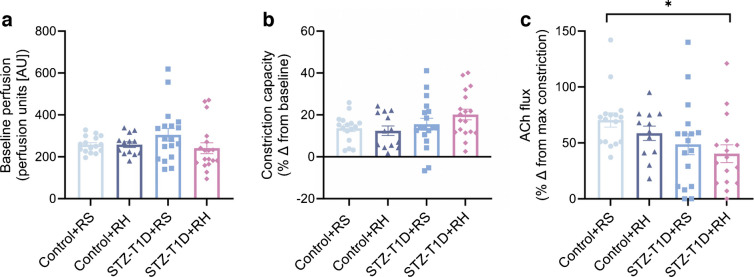
After 6 weeks, recurrent hypoglycaemia exacerbates microvascular endothelial dysfunction associated with hyperglycaemia. (**a**, **b**) Assessment of in vivo microvascular function via laser Doppler imaging coupled with iontophoresis of vasoactive compounds demonstrated no impact on resting blood flow (**a**) or vasoconstriction in response to phenylephrine (**b**). In contrast, recurrent hypoglycaemia exacerbates the effects of hyperglycaemia-driven endothelial dysfunction in response to transdermal ACh application after 6 weeks of exposure (**c**). Data are presented as mean ± SEM. Statistical significance determined by two-way ANOVA with Tukey’s post hoc analysis: *p<0.05 (*n*=12–18 mice per group)

Laser Doppler imaging assessment of peripheral microvascular endothelial function revealed no change in resting blood flow (Fig. 2a) or vasoconstriction in response to phenylephrine (Fig. 2b) after 6 weeks in any of the mouse models. A significant reduction in endothelial-dependent vasodilation was observed in response to ACh application in STZ-T1D mice compared with control mice (main effect of diabetes; *p*<0.05). Post hoc analysis revealed that recurrent hypoglycaemia augments this deficit (STZ-T1D+RH vs Control+RS, *p*<0.05; Fig. 2c). There was no significant difference between STZ-T1D+RS and STZ-T1D+RH mice nor between Control+RS and Control+RH mice; however, a 17% reduction in microvascular endothelial function was noted between Control+RS and Control+RH mice (*p*=0.32).

### Recurrent hypoglycaemia promotes a functional compensatory response in the left ventricle in non-diabetic mice

**Fig. 3:**
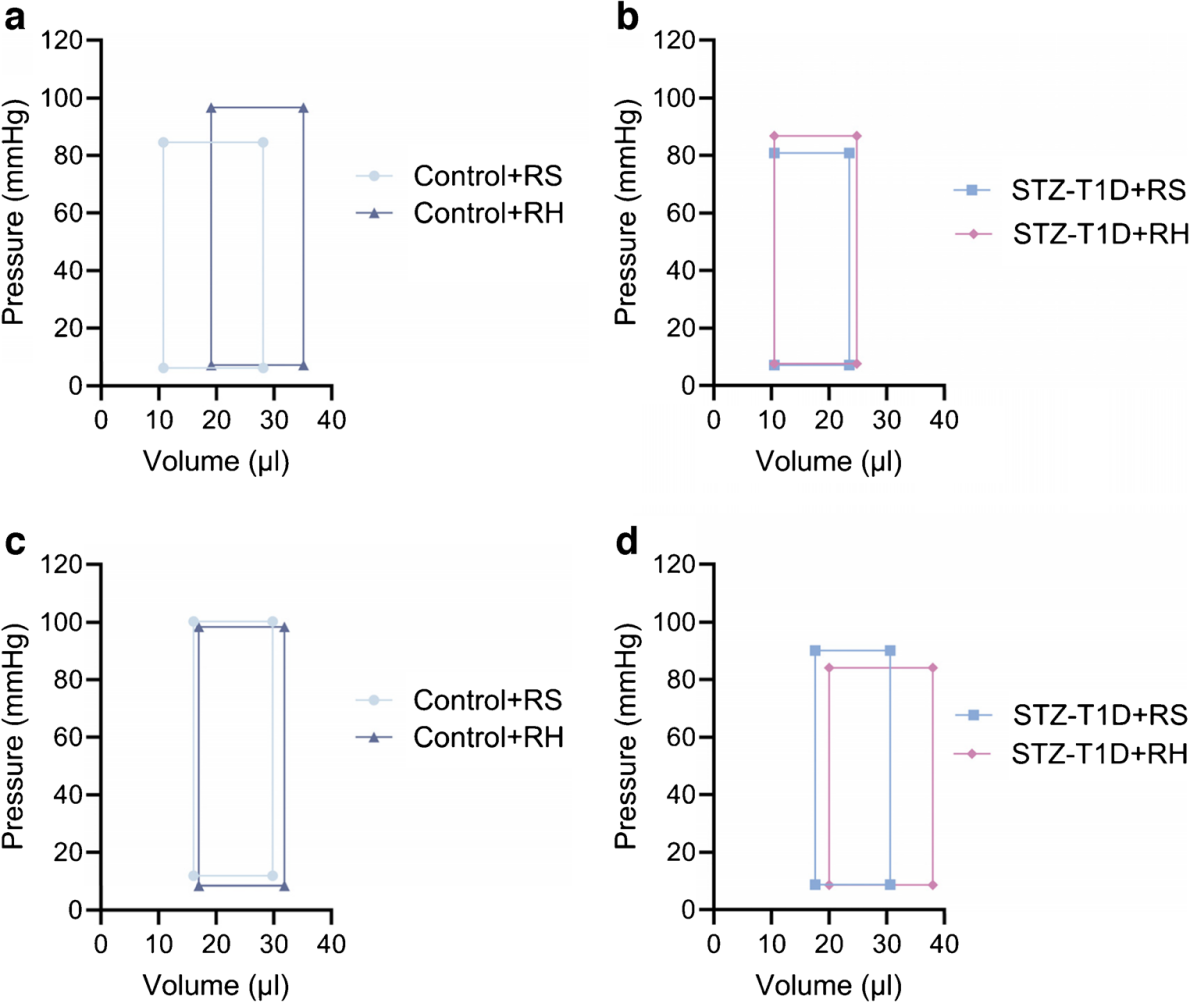
Representative PV loops of the left ventricle depicting the impact of diabetes and recurrent hypoglycaemia at the 6 week and 20 week time points. (**a**, **b**) After 6 weeks of recurrent hypoglycaemia, Control+RH mice show an elongated (tall) loop with a shift to the right (**a**), whereas no discernible difference was apparent between STZ-T1D+RS- and STZ-T1D+RH-treated mice (**b**). (**c**, **d**) However, after 20 weeks’ exposure to recurrent hypoglycaemia, the difference between Control+RS and Control+RH mice was lost (**c**) and a deficit in cardiac function in STZ-T1D+RH mice was seen (**d**), represented by the shortening and widening of the loop with a simultaneous rightward shunt, indicative of progression towards heart failure. The data supporting these figures are shown in Tables 1 and 2. Data are presented as mean ± SEM (*n*=per group stated in Tables 1 and 2)

Left ventricular function was assessed using PV loops at 6 and 20 weeks. Coupled with a small decrease in peripheral vascular tone (*p*=0.32), recurrent hypoglycaemia exposure in control animals resulted in elevated end-systolic pressure and a significant increase in end-systolic volume (effect of recurrent hypoglycaemia: *p*<0.01) at 6 weeks, while overall indices of performance remained unchanged (Table 1, Fig. 3a). Importantly, these differences were not observed by week 20 (Table 2, Fig. 3c), highlighting an initial ability to compensate and maintain function under greater pressures at week 6 before adaptation, at least under euglycaemic conditions, by week 20. Although no differences were observed in cardiac output, stroke work or arterial elastance in response to hypoglycaemia in control animals at week 6 (Table 1), Control+RH mice had impaired ventricular emptying compared with Control+RS mice, illustrated by a significant decrease in ejection fraction (*p*<0.05) and reduction in preload-adjusted maximum change of pressure over time (dP/dT_max_/EDV: 372.7 ± 61.38 vs 280.3 ± 30.95) at 6 weeks. Conversely, indices of filling (minimum rate of pressure rise [dP/dT_min_] and end-diastolic volume were elevated following 6 weeks of recurrent hypoglycaemia in control animals (effect of recurrent hypoglycaemia: *p*<0.05 for both), indicating a larger interventricular area and a more relaxed and dilated ventricle at week 6, which was not evident by week 20.

**Table 1:** PV loop analysis of left ventricular function at rest following 6 weeks of recurrent hypoglycaemia or saline treatment

Parameter	Control+RS	Control+RH	STZ-T1D+RS	STZ-T1D+RH
Aortic SBP (mmHg)	97.4 ± 3.83	97.4 ± 3.30	89.2 ± 3.41	96.0 ± 2.13
Aortic DBP (mmHg)	67.7 ± 3.51	68.1 ± 3.37	56.9 ± 3.75	63.8 ± 2.04
Heart rate (bpm)	543.0 ± 30.73	561.8 ± 21.26	566.0 ± 14.30	562.9 ± 9.47
Pressures				
End-systolic pressure (mmHg)	84.6 ± 3.38	96.7 ± 4.09*	80.8 ± 3.86 ‡	86.77 ± 2.91
End-diastolic pressure (mmHg)	6.2 ± 0.71	7.2 ± 1.69	7.1 ± 0.77	7.66 ± 1.24
dP/dT_max_ (mmHg/min)	9384.0 ± 786.4	9556.3 ± 1010.1	7453.8 ± 477.0	8821.2 ± 412.4^†^
dP/dT_min_ (mmHg/min)	−8617.3 ± 718.4	−10,306.4 ± 869.8^†^	−7164.5 ± 363.3	−8445.6 ± 494.7
Volumes				
End-systolic volume (µl)	10.8 ± 1.32	19.1 ± 1.54**	10.5 ± 1.31^‡‡^	10.5 ± 1.14^‡‡‡^
End-diastolic volume (µl)	28.1 ± 3.35	35.1 ± 2.58*	23.5 ± 1.65^‡‡^	24.8 ± 1.24^‡‡^
Stroke volume (µl)	22.1 ± 1.53	23.3 ± 1.25	16.3 ± 0.71**^,‡‡‡^	17.2 ± 0.59**^,‡‡‡^
Cardiac performance				
Ejection fraction (%)	71.1 ± 3.33	59.0 ± 2.15*	64.4 ± 2.94	65.7 ± 2.47
Cardiac output (µl min^–1^ g^–1^)	125.7 ± 14.5	126.8 ± 9.96	126.3 ± 4.32	136.5 ± 5.71
Stroke work (mmHg µl^–1^ g^–1^)	21.3 ± 2.49	22.2 ± 1.94	18.1 ± 1.12	21.2 ± 1.12
Arterial elastance (mmHg/µl)	3.9 ± 0.24	4.3 ± 0.22	5.1 ± 0.302	5.2 ± 0.29
Tau-Weiss (ms)	6.0 ± 0.47	5.3 ± 0.37	6.3 ± 0.338	6.6 ± 0.59
Load independent				
ESPVR	5.5 ± 0.59	6.2 ± 0.49	6.4 ± 0.33	7.0 ± 0.46
EDPVR	0.193 ± 0.021	0.240 ± 0.045	0.243 ± 0.030	0.299 ± 0.036
dP/dT_max_/EDV	372.7 ± 61.38	280.3 ± 30.95	332.2 ± 33.63	370.0 ± 24.56
Heart weight (mg)	135.2 ± 7.0	142.6 ± 4.9	104.4 ± 4.9***^,‡‡‡^	102.1 ± 2.7***^,‡‡‡^
LV weight (mg)	99.5 ± 5.5	104.9 ± 4.1	75.5 ± 3.6***^,‡‡‡^	73.0 ± 1.9***^,‡‡‡^
Body weight (g)	31.0 ± 0.8	31.1 ± 0.7	26.3 ± 0.8***^,‡‡‡^	26.3 ± 0.6***^,‡‡‡^
Weight change (g)	2.8 ± 0.8	2.3 ± 0.5	−0.1 ± 0.6***^,‡‡‡^	0.8 ± 0.3*
Number	6	7	8	15

The main effects of diabetes and recurrent hypoglycaemia are described in the text and post hoc analysis, highlighting significant differences as follows: *p<0.05 vs Control+RS, ^†^*p*<0.05 vs STZ-T1D+RS, ^‡^*p*<0.05 vs Control+RH; *p<0.05, **p<0.01, ***p<0.001 (or alternative symbol) determined by two-way ANOVA with Tukey’s post hoc analysis

DBP, diastolic blood pressure; dP/dT_max_, maximum rate of pressure rise; dP/dT_max_/EDV, preload-adjusted maximum change of pressure over time; dP/dT_min_, minimum rate of pressure rise; EDPVR, end-diastolic pressure–volume relationship; ESPVR, end-systolic pressure–volume relationship; LV, left ventricular; SBP, systolic blood pressure

**Table 2:** PV loop analysis of left ventricular function at rest following 20 weeks of recurrent hypoglycaemia or saline treatment

Parameter	Control+RS	Control+RH	STZ-T1D+RS	STZ-T1D+RH
Aortic SBP (mmHg)	102.6 ± 7.0	101.9 ± 3.1	99.0 ± 6.0	91.9 ± 3.8
Aortic DBP (mmHg)	66.3 ± 5.8	64.8 ± 2.1	62.5 ± 3.9	61.3 ± 2.3
Heart rate (bpm)	558.1 ± 13.6	518.7 ± 25.2	500.1 ± 15.7	509.3 ± 11.0
Pressures				
End-systolic pressure (mmHg)	100.3 ± 6.5	98.4 ± 3.9	90.1 ± 5.8*	84.1 ± 3.8*
End-diastolic pressure (mmHg)	11.8 ± 2.1	8.4 ± 1.2	8.7 ± 1.4	8.6 ± 1.8
dP/dT_max_ (mmHg/min)	8811.2 ± 520.7	9598.4 ± 502.7**	6782.3 ± 551.5	6534.3 ± 447.2*^,‡‡^
dP/dT_min_ (mmHg/min)	−7449.3 ± 388.0	−9559.6 ± 683.0*	−6866.3 ± 491.3	−6683.7 ± 599.5^‡^
Volumes				
End-systolic volume (µl)	16.1 ± 1.8	17.0 ± 2.4	17.6 ± 1.1	20.0 ± 2.6
End-diastolic volume (µl)	29.8 ± 1.1	31.9 ± 2.1	30.6 ± 2.4	38.0 ± 1.7*
Stroke volume (µl)	15.5 ± 1.5	17.7 ± 1.1	14.5 ± 1.8	21.2 ± 2.7
Cardiac performance				
Ejection fraction (%)	50.3 ± 5.3	55.0 ± 4.4	45.6 ± 3.1	52.9 ± 5.7
Cardiac output (µl min^–1^ g^–1^)	88.4 ± 16.3	98.3 ± 16.4	84.6 ± 9.9	131.6 ± 16.2^†^
Stroke work (mmHg µl^–1^ g^–1^)	14.0 ± 1.8	18.4 ± 2.3	14.4 ± 1.8	20.9 ± 3.0
Arterial elastance (mmHg/µl)	7.0 ± 0.9	5.7 ± 0.4	6.9 ± 1.1	4.4 ± 0.6
Tau-Weiss (ms)	7.3 ± 0.7	6.0 ± 0.4	7.4 ± 0.6	7.4 ± 0.8
Load independent				
ESPVR	6.3 ± 0.3	4.5 ± 0.5	6.3 ± 0.4	5.6 ± 0.6
EDPVR	0.27 ± 0.06	0.24 ± 0.03	0.30 ± 0.05	0.29 ± 0.04
dP/dT_max_/EDV	299.6 ± 24.5	316.9 ± 38.6	225.3 ± 15.5	173.5 ± 11.7*^,‡‡^
Heart weight (mg)	149.1 ± 9.1	140.2 ± 4.3	115.9 ± 3.0**^,‡^	115.8 ± 3.0**^,‡^
LV weight (mg)	104.1 ± 5.4	98.1 ± 5.3	83.5 ± 2.8*	81.2 ± 2.5**^,‡^
Body weight (g)	41.2 ± 2.4	43.3 ± 1.1	28.4 ± 0.6***^,‡‡‡^	29.2 ± 0.4***^,‡‡‡^
Weight change (g)	2.7 ± 0.43	4.6 ± 0.3	−4.1 ± −0.5***^,‡‡‡^	−0.8 ± 0.2^‡‡^
Number	6	7	6	7

The main effects of diabetes and recurrent hypoglycaemia are described in the text and post hoc analysis, highlighting significant differences as follows: *p<0.05 vs Control+RS, ^†^*p*<0.05 vs STZ-T1D+RS, ^‡^*p*<0.05 vs Control+RH; *p<0.05, **p<0.01, ***p<0.001 (or alternative symbol) determined by two-way ANOVA with Tukey’s post hoc analysis

DBP, diastolic blood pressure; dP/dT_max_, maximum rate of pressure rise; dP/dT_max_/EDV, preload-adjusted maximum change of pressure over time; dP/dT_min_, minimum rate of pressure rise; EDPVR, end-diastolic pressure–volume relationship; ESPVR, end-systolic pressure–volume relationship; LV, left ventricular; SBP, systolic blood pressure

### Chronic hyperglycaemia impairs compensatory adaptation to recurrent hypoglycaemia and promotes left ventricular dilation

We then examined cardiac parameters and whether the presence of chronic hyperglycaemia in STZ-T1D mice altered these compensatory adaptations to recurrent hypoglycaemia. Consistent with their lower body weight, STZ-T1D mice had smaller hearts at 6 weeks than non-diabetic control mice (effect of diabetes: *p*<0.001), associated with significantly impaired load-dependent parameters of cardiac function (stroke volume, end-diastolic volume, maximum rate of pressure rise dP/dT_min_; Table 1). Differences persisted at 20 weeks, with both STZ-T1D groups having smaller body weights (effect of diabetes: *p*<0.001) and heart weights (effect of diabetes: *p*<0.01), as well as lower end-systolic pressures (effect of diabetes: *p*<0.05) than their non-diabetic counterparts (Table 2). Diabetic hearts did not display evidence of compensatory function after 6 weeks of recurrent hypoglycaemia exposure (Fig. 3b). However, although indices of performance were maintained at week 20, prolonged recurrent hypoglycaemia exposure appeared to promote characteristic shifts associated with diabetic cardiomyopathy, ventricular dilation and early heart failure, including an elevated end-diastolic volume (effect of diabetes: *p*<0.05) in the absence of left ventricular hypertrophy (unchanged left ventricular mass; effect of recurrent hypoglycaemia: *p*=0.93). This shift towards an early heart failure-like phenotype is visible as a widening and downward shift in the cardiac cycle (Fig. 3d) and in a larger cardiac output and stroke work (effect of recurrent hypoglycaemia: *p*<0.05 for both), with preserved ejection fraction in STZ-T1D+RH mice compared with STZ-T1D+RS mice (Table 2). Load-independent contractility, as measured by dP/dT_max_/EDV, was significantly impaired following 20 weeks of recurrent hypoglycaemia exposure in STZ-T1D+RH mice (*p*<0.01), suggesting a defective systolic response to altered preload. Importantly, these effects appeared to be uniquely influenced by the combination of hyper- and hypoglycaemia and were not significantly influenced by hyperglycaemia alone at 20 weeks.

### Cardiac performance under hypoglycaemic stress

**Fig. 4:**
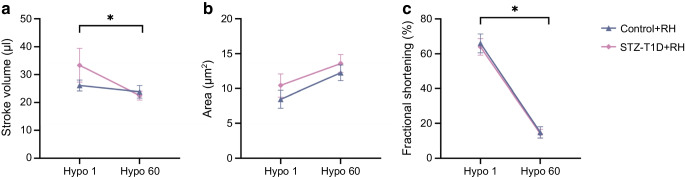
Ultrasound imaging via echocardiography under hypoglycaemic challenge reveals distinct cardiac responses to hypoglycaemia exposure following prior long-term recurrent hypoglycaemic exposure that are not observed on first exposure to hypoglycaemia. Comparative analysis of heart function during acute (Hypo 1) vs recurrent hypoglycaemic (Hypo 60) stress performed using mixed-model ANOVA, identified functional adaptation in stroke volume (**a**) and fractional shortening (**c**), with a non-significant increase in internal ventricular area also observed (**b**, *p*=0.0604), following prior repeated exposure. Data are presented as mean ± SEM. Statistical significance determined by two-tailed *t* test: *p<0.05 (*n*=3–13)

To assess cardiac function longitudinally, echocardiography was performed prior to and following recurrent hypoglycaemia exposure (control and post-STZ, weeks 1 and 20), including responses during the first (acute) and 60th exposure to hypoglycaemia (recurrent), in separate cohorts of mice (ESM Table 2, Fig. 4). No significant differences in cardiac function were found between groups at rest (ESM Table 2). In line with the PV loop experiments, the internal ventricular area increased between the first and 60th hypoglycaemic episodes, although this occurred regardless of the diabetic background (*p*=0.06; Fig. 4b). However, stroke volume (effect of recurrent hypoglycaemia: *p*<0.05; Fig. 4a) and fractional shortening (effect of recurrent hypoglycaemia: *p*<0.05; Fig. 4c) measured during hypoglycaemia were significantly reduced in both Control+RH and STZ-T1D+RH mice following recurrent hypoglycaemia.

### Recurrent hypoglycaemia alters the expression of genes associated with cardiac electrochemical activity within left ventricular tissue

**Fig. 5:**
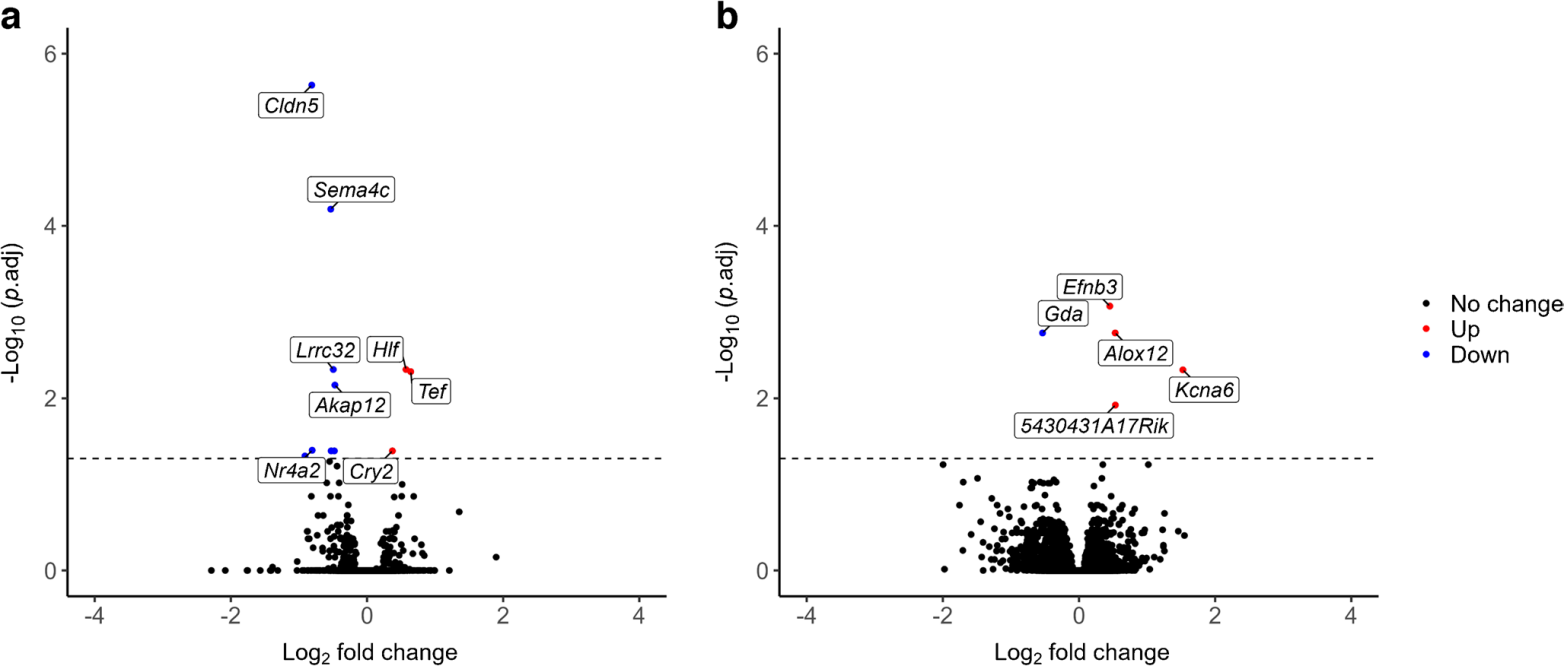
Recurrent hypoglycaemia regulates expression of genes involved in cardiac electrochemical activity and endothelial health. Bulk RNA-seq of left ventricular tissue from mice exposed to 20 weeks of experimental hypoglycaemia identified 11 differentially expressed genes (DEGs) when comparing Control+RS and Control+RH mice (**a**). A full list of these DEGs is provided in Table 3. When comparing STZ-T1D+RS and STZ-T1D+RH mice, only five DEGs were identified (**b**); however, these genes have functional roles linked to cardiomyocyte membrane repolarisation. A full list of these genes is provided in Table 4. DEGs were classed as genes with *p*(adj)<0.05 and log_2_ fold change <0 or >0. The dotted line intersects the y-axis at –log_10_(1.3), which is equivalent to *p*(adj)=0.05

Bulk RNA-seq of left ventricular tissue (*n*=16; *n*=4 per group) was performed following completion of the 20 week study. Following quality control assessment, one sample was removed from the Control+RH group. Upregulation of gene expression was greatest in STZ-T1D mice vs control mice. KEGG analysis revealed that transcription shifts in response to chronic hyperglycaemia were associated with immune processes and cardiomyopathy (ESM Fig. 2e,f). Recurrent hypoglycaemia in the absence of diabetes was associated with changes in a small subset of genes associated with endothelial integrity, β-adrenergic signalling and heart failure, including *Cldn5*, *Akap12*, *Nr4a2* and *Adamts4* (Fig. 5a, Table 3). Additionally, downregulation of the Mg^2+^ efflux-associated gene *Slc41a3* was observed in Control+RH mice. In STZ-T1D mice, only *Gda*, a gene associated with xanthine production and inhibition of coronary microvascular vasodilation, was downregulated by recurrent hypoglycaemia. There were four upregulated genes; three are associated with progression or severity of heart failure (*Efnb3*, *Alox12* and *5430431a17Rik*), whereas *Kcna6* encodes for a pore-forming subunit of the Kv1 voltage-gated potassium transporter involved in membrane repolarisation during the cardiac action potential and prolongation of the QT interval (STZ-T1D+RS vs STZ-T1D+RH; Fig. 5b, Table 4).

**Table 3:** Differentially expressed genes identified by RNA-seq analysis of left ventricular tissue from Control+RS vs Control+RH mice

Gene ID	Gene name	Log_2_ fold change	*p*(adj)
*Cldn5*	Claudin 5	−0.81201	2.32 × 10^–6^
*Sema4c*	Semaphorin 4C	−0.53298	6.41 × 10^–5^
*Hlf*	Hepatic leukaemia factor	0.572664	0.004622
*Lrrc32*	Leucine rich repeat containing 32	−0.49566	0.004622
*Tef*	Thyrotroph embryonic factor	0.642032	0.004899
*Akap12*	A-kinase anchoring protein 12	−0.47143	0.007019
*Nr4a2*	Nuclear receptor subfamily 4 group A member 2	−0.80575	0.040122
*Slc41a3*	Solute carrier family 41 member 3	−0.48034	0.040798
*Trp53i11*	Tumour protein p53 inducible protein 11	−0.52688	0.040798
*Cry2*	Cryptochrome circadian regulator 2	0.373104	0.040798
*Adamts4*	ADAM metallopeptidase with thrombospondin type 1 motif 4	−0.91189	0.046783

**Table 4:** Differentially expressed genes identified by RNA-seq analysis of left ventricular tissue from STZ-T1D+RS vs STZ-T1D+RH mice

Gene ID	Gene name	Log_2_ fold change	*p*(adj)
*Efnb3*	Ephrin B3	0.452772	0.000857
*Alox12*	Arachidonate 12-lipoxygenase	0.532017	0.001758
*Gda*	Guanine deaminase	−0.53515	0.001758
*Kcna6*	Potassium voltage-gated channel subfamily A member 6	1.526579	0.00467
*5430431A17Rik*	lncRNA	0.534392	0.011974

## Discussion

Using a type 1 diabetes mouse model, we examined how recurrent hypoglycaemia affects microvascular function, cardiac haemodynamics and cardiac tissue. Chronic hyperglycaemia impaired microvascular endothelial function, which was further worsened by recurrent hypoglycaemia at clinically relevant frequencies. In non-diabetic animals, 20 weeks of sustained exposure to recurrent hypoglycaemia promoted a compensatory shift in cardiac function; this was not mirrored in type 1 diabetic animals, who progressed towards ventricular dilation, cardiomyopathy and early heart failure. We also report that, in both type 1 diabetic and non-diabetic mice, cardiac systolic function during subsequent hypoglycaemia was significantly impaired in those exposed to recurrent hypoglycaemia, indicating reduced cardiac flexibility. Transcriptomic analysis of cardiac tissue indicated that these changes may result from impaired left ventricular remodelling, as well as highlighting a potential increased susceptibility to cardiac arrhythmias.

Epidemiological studies of people with diabetes often rely on retrospective severe (level 3) hypoglycaemia data to assess effects on microvascular and cardiac function. However, for the average person with type 1 diabetes, severe events are infrequent (0.3–3.2/year), compared with >100 annual total hypoglycaemic episodes [11, 26, 27]. To address this, we used a rodent STZ-induced type 1 diabetes model with basal insulin replacement and controlled recurrent hypoglycaemia, which allows better modelling of the human experience of hypoglycaemia and permits in vivo and ex vivo analysis. In our model, basal insulin replacement was provided by subcutaneous insulin implants (LinBit), which we have shown previously are sufficient to prevent metabolic decompensation [19], while recurrent hypoglycaemia was induced at planned intervals with rapid-acting insulin. It should be noted that this model does not replicate intensive insulin therapy with multiple daily insulin injections but does allow for a more controlled assessment of the independent effects of chronic hyperglycaemia and recurrent hypoglycaemia on cardiac function and any interaction between the two metabolic states.

### Hyperglycaemia-driven inflexibility

The cardiovascular effects of hyperglycaemia involve mitochondrial overload, ROS production, inflammation and tissue damage [6]. Endothelial dysfunction arises from increased endothelin-1, reduced endothelial nitric oxide synthase (eNOS) activity and lower NO bioavailability, promoting vasoconstriction, hypertension, and left ventricular hypertrophy [28–31]. In our model, chronic hyperglycaemia impaired microvascular endothelial function after 6 weeks. Recurrent hypoglycaemia had no effect in non-diabetic mice but exacerbated endothelial dysfunction in type 1 diabetic mice (Fig. 2c). This suggests that recurrent hypoglycaemia may either exacerbate the action of chronic hyperglycaemia to suppress endothelial generation of NO in response to ACh or disrupt the translocation or action of endothelial-derived vasodilators on the vascular smooth muscle. Our data support previous literature showing that antecedent hypoglycaemia, particularly when followed by hyperglycaemia, impairs NO-dependent vasodilation in people with and without diabetes [28].

The heart adapts to altered physiology, often at the expense of longevity. Six weeks of recurrent hypoglycaemia in non-diabetic mice induced compensatory changes compared with control mice (Control+RS), maintaining normal cardiac function (Table 1, Fig. 3). By 20 weeks, cardiac volumes were similar between control non-diabetic mice and non-diabetic mice exposed to recurrent hypoglycaemia, suggesting further adaptations over time (Table 2, Fig. 3). In type 1 diabetic mice, 6 weeks of chronic hyperglycaemia led to smaller hearts with reduced volumes and stroke volumes (Table 2), consistent with a blunted weight trajectory. After 20 weeks of recurrent hypoglycaemia, no differences in cardiac performance were apparent between non-diabetic and type 1 diabetic mice. In contrast, STZ-T1D+RH mice exhibited increased cardiac output (*p*<0.05) and stroke volumes (*p*<0.05) compared with their STZ-T1D littermates, despite having smaller hearts and left ventricular weights compared with non-diabetic mice. The load-independent rate at which the ventricle can reach maximum pressure (dP/dTmax/EDV) was significantly impaired in STZ-T1D+RH mice, consistent with developing systolic dysfunction at rest. Taken together, these results indicate that STZ-T1D+RH mice experience ventricular dilation and early dilated cardiomyopathy.

### Recurrent hypoglycaemia-induced cardiovascular ‘vulnerability’

PV loop studies demonstrated that recurrent hypoglycaemia-driven cardiac adaptations maintain basal function. However, echocardiography revealed reduced stroke volume and fractional shortening during the 60th vs first hypoglycaemic exposure, indicating that systolic dysfunction emerges during a subsequent hypoglycaemic challenge (Fig. 4). While ageing may contribute, recurrent hypoglycaemia-exposed animals displayed impaired function, which, coupled with structural adaptations at rest observed in STZ-T1D+RH mice, could render the heart more vulnerable to alternate stressors, possibly contributing to increased risk of a cardiovascular event and/or poorer survival from cardiovascular events in people with type 1 diabetes [29]. Further investigation of this effect is warranted.

Recurrent hypoglycaemia may promote chronic dysfunction via repeated damage and remodelling, as seen in our vascular and PV loop data (Figs 2, 3). In people with type 1 and type 2 diabetes and in those without diabetes, acute hypoglycaemia triggers prolonged systemic inflammation lasting up to a week, linked to oxidative stress [14, 30, 31]. Post-hypoglycaemic hyperglycaemia induces additional oxidative stress and inflammation [32]. In animal models of type 1 diabetes, the ‘double hit’ of hypo- and post-hypoglycaemic hyperglycaemia results in cognitive dysfunction and oxidative damage in brain tissue [18, 19]. Therefore, increased glycaemic variability resulting from the combination of chronic hyperglycaemia and post-hypoglycaemic hyperglycaemia may lead to an increased oxidative and inflammatory burden, exacerbating endothelial dysfunction.

Both hyper- and hypoglycaemia independently increase inflammation, likely contributing to CVD progression. This was investigated further using RNA-seq of left ventricular tissue. KEGG pathway analysis highlighted the role of inflammation and altered immune activity in hyperglycaemic hearts (ESM Fig. 3). Recurrent hypoglycaemia produced no additional ventricular inflammation, possibly due to the timing of tissue harvest or tissue-specific effects. Recurrent hypoglycaemia-associated inflammation has been reported in the brain [19] but remains to be assessed in the heart and peripheral vasculature.

### Recurrent hypoglycaemia alters the expression of genes associated with electrochemical gradient homeostasis and repolarisation

Bulk RNA-seq revealed transcriptomic changes associated with hyperglycaemia and recurrent hypoglycaemia. In STZ-T1D+RH mice, *Kcna6* (encoding potassium voltage-gated channel subfamily A member 6) was upregulated. Potassium channels have been linked to cardiac dysrhythmias during hypoglycaemia [33]. Further research is needed to understand the functional significance of this observation and whether the elevated expression of *Kcna6* corresponds to an increased membrane presence of the Kv1.6 channel, and subsequently, how this impacts membrane potential and repolarisation during physiological or metabolic stress. Recurrent hypoglycaemia also downregulated *Slc41a3*, which encodes a Mg^2+^ exchanger that has previously been implicated in action potential regulation [34]. Several studies have shown that hypoglycaemia disrupts cardiac action potentials in rodents [35, 36]. Future studies should explore these ‘channelopathies’ using ECG, echocardiography and targeted interventions.

### Limitations

The limitations of this study include the use of STZ to induce diabetes. STZ mimics glucose and is transported into cells through the GLUT2 transporter. GLUT2 is not expressed in the heart, but acute administration of high doses of STZ induces cardiomyocyte death and dysfunction. Additionally, despite careful limitation of exposure and anaesthesia depth, isoflurane may affect haemodynamics. Finally, caution should be taken when applying preclinical cardiovascular studies in rodents to humans, as disease pathology can vary significantly.

### Conclusion

In conclusion, we report that, in a mouse model of type 1 diabetes, recurrent hypoglycaemia promotes microvascular dysfunction with compensatory shifts in cardiac haemodynamics indicative of early dilated cardiomyopathy, increasing cardiac vulnerability. Transcriptomic changes associated with heart failure and membrane polarisation in response to recurrent hypoglycaemia suggest potential mechanistic pathways underlying increased cardiac vulnerability, which could form the basis for future therapeutic investigation.

## Supplementary Information

Below is the link to the electronic supplementary material.

ESM (PDF 628 KB)

ESM Data File (ZIP 72.0 MB)

## Data Availability

Raw data are available on request to the corresponding authors. Transcriptomic data are provided in the ESM Data File.

## Abbreviations

ACh: Acetylcholine
bpm: Beats per minute
Control+RH: Non-diabetic mice treated with recurrent hypoglycaemia
Control+RS: Non-diabetic mice treated with recurrent saline
dP/dT_max_: Maximum rate of pressure rise
dP/dT_max_/EDV: Preload-adjusted maximum change of pressure over time
dP/dT_min_: Minimum rate of pressure rise
KEGG: Kyoto Encyclopaedia of Genes and Genomics
NO: Nitric oxide
PV: Pressure–volume
ROS: Reactive oxygen species
STZ: Streptozocin
STZ-T1D+RH: Type 1 diabetic mice treated with recurrent hypoglycaemia
STZ-T1D+RS: Type 1 diabetic mice treated with recurrent saline
