# Supplementary material for: Recurrent hypoglycaemia promotes cardiomyopathy and cardiac vulnerability in a rodent model of type 1 diabetes
Source: Diabetologia. 2025 Nov 10;69(2):515–28. doi: 10.1007/s00125-025-06574-5 (PMC12779658; doi:10.1007/s00125-025-06574-5)
Supplement: Supplementary file 1 — ESM (PDF 628 KB) [file 125_2025_6574_MOESM1_ESM.pdf]

ESM methods

Supplementary Methodology

Left Ventricle RNA-seq

Tissue Dissection and RNA extraction

Left ventricular tissue was carefully separated from the whole heart immediately after perfusion with KCl and further dissected into three distinct regions (upper (proximal ventricle and aortic root), middle and lower (apex). To accurately investigate the transcriptome of contractile cardiomyocytes and minimise contamination by non-contractile tissue (valves, adventitia), 40mg of 'middle' LV was isolated for RNA extraction via TRIzol (Invitrogen, Carlsbad, CA) using vortex bead homogenisation according to the manufacturer's instructions. The resulting RNA was quantified, purity assessed (Thermofisher Nanodrop), flash frozen in liquid nitrogen and stored on dry ice during transportation to AZENTA Life Sciences for sequencing. The following protocol was kindly provided by AZENTA Life Sciences.

RNA Library Preparation and NovaSeq Sequencing

RNA samples were quantified using a Qubit 4.0 Fluorometer (Life Technologies, Carlsbad, CA, USA). RNA integrity was checked with an RNA Kit on Agilent 5300 Fragment Analyzer (Agilent Technologies, Palo Alto, CA, USA).

RNA sequencing libraries were prepared using the NEBNext Ultra II RNA Library Prep Kit for Illumina following the manufacturer's instructions (NEB, Ipswich, MA, USA). Briefly, mRNAs were first enriched with Oligo(dT) beads. Enriched mRNAs were fragmented according to the manufacturer's instructions. First-strand and second-strand cDNAs were subsequently synthesised. cDNA fragments were end-repaired and adenylated at 3'ends, and universal adapters were ligated to cDNA fragments, followed by index addition and library enrichment by limited-cycle PCR. Sequencing libraries were validated using NGS Kit on the Agilent 5300 Fragment Analyzer (Agilent Technologies, Palo Alto, CA, USA) and quantified using Qubit 4.0 Fluorometer (Invitrogen, Carlsbad, CA). The sequencing libraries were multiplexed and loaded on the flow cell on the Illumina NovaSeq 6000 instrument according to the manufacturer's instructions. The samples were sequenced using a 2x150 Pair-End (PE) configuration

v1.5. The NovaSeq Control Software v1.7 conducted image analysis and base calling. Raw sequence data (.bcl files) generated from Illumina NovaSeq was converted into fastq files and de-multiplexed using Illumina bcl2fastq program version 2.20. One mismatch was allowed for index sequence identification.

DESeq2 RNA-seq Data Analysis

After investigating the quality of the raw data, sequence reads were trimmed to remove adapter sequences and nucleotides with poor quality using Trimmomatic v.0.36. The trimmed reads were mapped to the *Mus musculus* reference genome available on ENSEMBL using the STAR aligner v.2.5.2b. BAM files were generated because of this step. Unique gene hit counts were calculated using feature Counts from the Subread package v.1.5.2. Only unique reads that fell within exon regions were counted.

After extracting the gene hit counts, the gene hit counts table was used for downstream differential expression analysis. Using DESeq2, a comparison of gene expression between the groups of samples was performed. The Wald test was used to generate P values and Log2 fold changes. Genes with adjusted P values < 0.05 and absolute log2 fold changes >0 were called differentially expressed for each comparison.

RNA-seq Downstream Data Analysis and Visualisation

Gene ontology (GO) and KEGG pathway enrichment analysis was performed on the statistically significant set of genes using the ClusterProfiler package in RStudio. RStudio package ggplot2 was used for data visualisation.

Major Resources

Unless stated otherwise, all consumables were sourced from Merck-Sigma®.

ESM Table 1: Cohort 3 Phenotyping data (20-week long-term model for echocardiography). Weight change (Week 0 vs. 20), heart & LV weights, mean resting glucose, mean hypo glucose ± SEM.

	**Control+RS**	**Control+RH**	**STZ-T1D+RS**	**STZ-T1D+RH**
**Weight change (g)**	5.1 ±0.65	4.0 ±0.81	0.2 ±0.48*** ‡‡‡	0.5 ±0.27*** ‡‡‡
**Heart weight (mg)**	146.6 ±5.28	131 ±3.21* ††	114.5 ±3.60***	113.4 ±1.71*** ‡‡
**LV Weight (mg)**	105.6 ±4.53	94.9 ±2.44†	82.8 ±2.41***	78.0 ±1.92*** ‡‡‡
**Mean Blood Glucose (mmol/l)**	10.0 ±0.35	9.5 ±0.18	28.3 ±1.26*** ‡‡‡	27.4 ±0.73*** ‡‡‡
**Mean Hypo Depth (mmol/l)**	9.9 ±0.34	2.6 ±0.06***	25.1 ±1.03*** ‡‡‡	2.9 ±0.06*** †††
**Number**	**9**	**12**	**13**	**12**

*vs Control+RS, †vs STZ-T1D+RS, ‡vs Control+RH ; *$p<0.05$, **$p<0.01$, ***$p<0.001$ (or alternative symbol) determined by two-way ANOVA with Tukey's post-hoc analysis.

ESM Table 2 - Cardiac Function assessed via echocardiography. Data presented as change from baseline (Week 20 - Week1) ± SEM. All parameters were calculated using B-mode, excluding heart rate which was determined from M-mode.

	**Control+RS**	**Control+RH**	**STZ-T1D+RS**	**STZ-T1D+RH**
End-systolic Volume *(mL)*	Δ1.81 ±2.19	Δ2.00 ±2.88	Δ0.16 ±2.34	Δ-0.27 ±2.15
End-diastolic Volume *(mL)*	Δ-2.24 ±4.54	Δ-0.15 ±3.77	Δ-7.14 ±4.13	Δ-1.63 ±5.19
Stroke Volume *(mL)*	Δ-4.04 ±3.45	Δ-2.14 ±2.55	Δ-7.29 ±2.71	Δ-1.36 ±3.62
Ejection Fraction *(%)*	Δ-8.32 ±4.96	Δ-5.38 ±5.21	Δ-5.17 ±4.06	Δ0.28 ±3.21
Cardiac Output *(ml/min/g)*	Δ-8.93 ±6.36	Δ-13.68 ±4.35	Δ-10.73 ±3.50	Δ-3.88 ±5.04
Fractional Shortening *(%)*	Δ-0.34 ±2.44	Δ-7.32 ±2.59	Δ5.50 ±4.45	Δ-0.37 ±4.31
Heart rate *(bpm)*	Δ15.15 ±29.94	Δ-14.17 ±17.68	Δ17.18 ±23.60	Δ3.19 ±18.86
Number	**12**	**12**	**12**	**13**

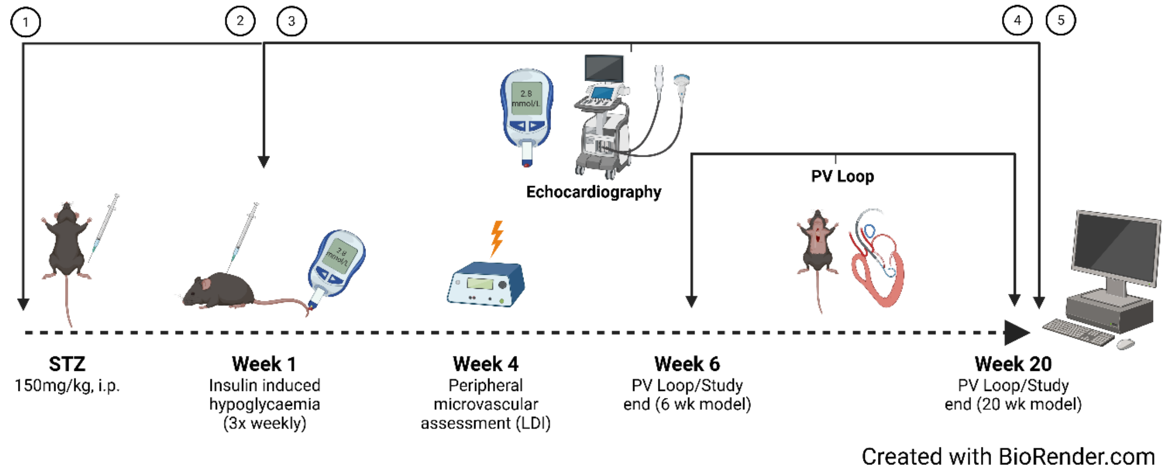

ESM Fig. 1 - Study design diagram including all 3 cohorts detailing procedure timelines for each cohort. Echocardiography was carried out at five distinct time points: 1 – pre-STZ, 2 – euglycaemic conditions (week 1), 3 – hypoglycaemic conditions (week 1), 4 – euglycaemic conditions (week 20) and 5 - hypoglycaemic conditions (week 20).

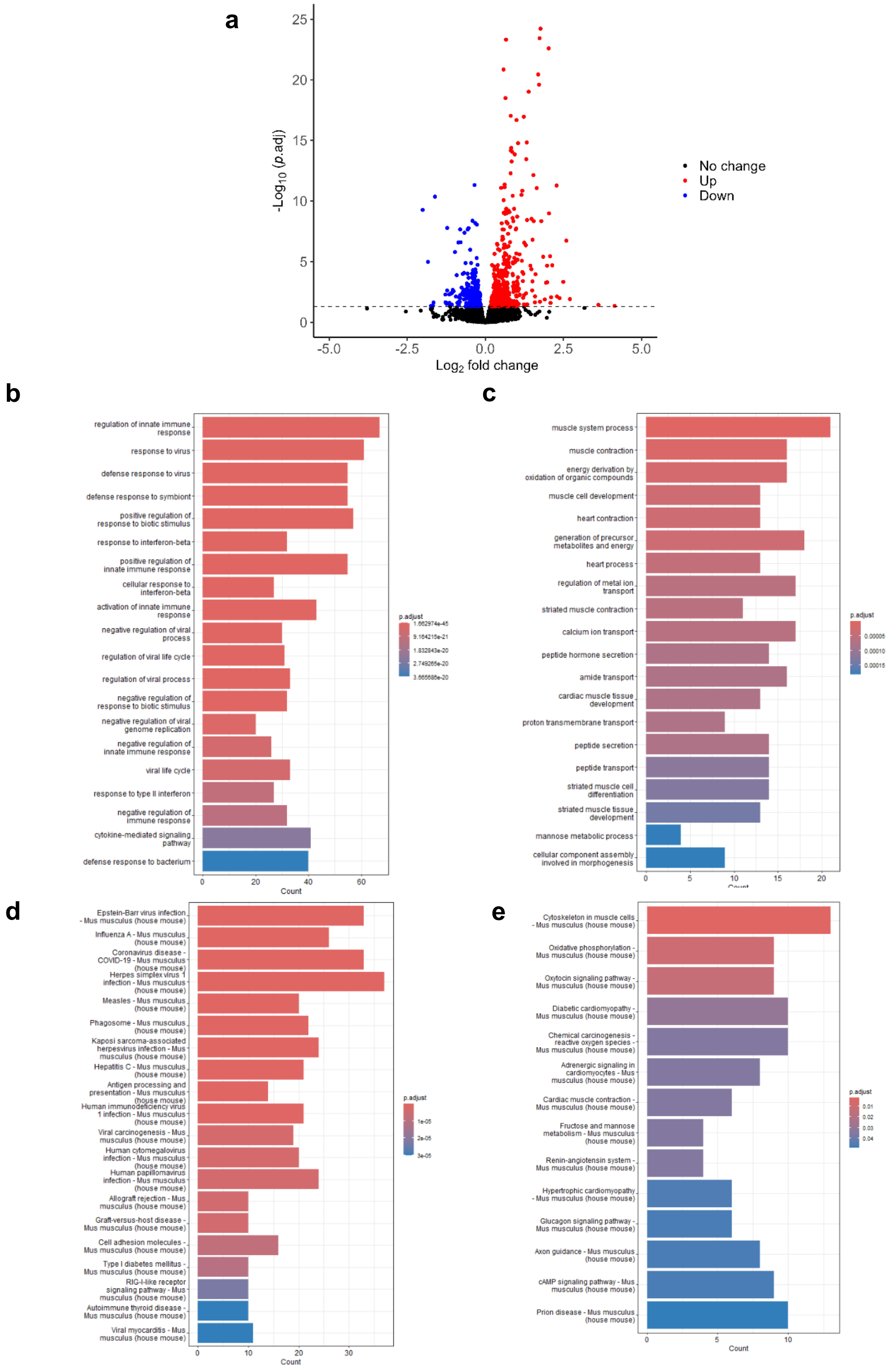

ESM Fig.2 – Left ventricle RNA sequencing data for Control+RS vs STZ-T1D+RS. (a) Volcano plot of differently expressed genes (DEGs were classed as genes with *p*(adj) < 0.05 and Log2 Fold Change < or > 0. The dotted line intersects the y-axis at –log10(1.3) which is equivalent to *p*(adj) = 0.05), (b) upregulated biological process GO terms, (c) downregulated biological process GO terms, (d) upregulated KEGG pathways and (f) downregulated KEGG pathways.
